# Genome-Wide DNA Methylation Analysis of a Cohort of 41 Patients Affected by Oculo-Auriculo-Vertebral Spectrum (OAVS)

**DOI:** 10.3390/ijms22031190

**Published:** 2021-01-26

**Authors:** Valentina Guida, Luciano Calzari, Maria Teresa Fadda, Francesca Piceci-Sparascio, Maria Cristina Digilio, Laura Bernardini, Francesco Brancati, Teresa Mattina, Daniela Melis, Francesca Forzano, Silvana Briuglia, Tommaso Mazza, Sebastiano Bianca, Enza Maria Valente, Leila Bagherjad Salehi, Paolo Prontera, Mario Pagnoni, Romano Tenconi, Bruno Dallapiccola, Giorgio Iannetti, Luigi Corsaro, Alessandro De Luca, Davide Gentilini

**Affiliations:** 1 Medical Genetics Division, Fondazione IRCCS Casa Sollievo della Sofferenza, San Giovanni Rotondo, 71013 Foggia, Italy; f.piceci@css-mendel.it (F.P.-S.); l.bernardini@css-mendel.it (L.B.); a.deluca@css-mendel.it (A.D.L.); 2 Istituto Auxologico Italiano IRCCS, Bioinformatics and Statistical Genomics Unit, Cusano Milanino, 20095 Milano, Italy; bisgu.auxologico@gmail.com; 3 Department of Maxillofacial Surgery, Sapienza University of Rome, 00161 Rome, Italy; mariateresa.fadda@uniroma1.it (M.T.F.); mariop77@gmail.com (M.P.); giorgio.iannetti@uniroma1.it (G.I.); 4 Department of Experimental Medicine, Sapienza University of Rome, 00161 Rome, Italy; 5 Genetics and Rare Diseases Research Division, Ospedale Pediatrico Bambino Gesù, IRCCS, 00165 Rome, Italy; mcristina.digilio@opbg.net (M.C.D.); bruno.dallapiccola@opbg.net (B.D.); 6 Department of Life, Health and Environmental Sciences, Unit of Medical Genetics University of L’Aquila, 67100 L’Aquila, Italy; francesco.brancati@univaq.it; 7 IRCCS San Raffaele Pisana, 00163 Rome, Italy; 8 Medical Genetics, Department of Biomedical and Biotechnological Sciences, University of Catania, 95131 Catania, Italy; mattina@unict.it; 9 Department of Medicine, Surgery and Dentistry, University of Salerno, 84084 Salerno, Italy; dmelis@unisa.it; 10 Clinical Genetics Department, Guy’s & St Thomas’ NHS Foundation Trust, London SE1 7EH, UK; Francesca.Forzano@gstt.nhs.uk; 11 Medical Genetics, University of Messina, 98125 Messina, Italy; sbriuglia@unime.it; 12 Unit of Bioinformatics, Fondazione IRCCS Casa Sollievo della Sofferenza, San Giovanni Rotondo, 71013 Foggia, Italy; t.mazza@css-mendel.it; 13 Centro di Consulenza Genetica e Teratologia della Riproduzione, Dipartimento Materno Infantile, ARNAS Garibaldi Nesima, 95123 Catania, Italy; sebastiano.bianca@tiscali.it; 14 Department of Molecular Medicine, University of Pavia, 27100 Pavia, Italy; enzamaria.valente@unipv.it; 15 IRCCS Mondino Foundation, 27100 Pavia, Italy; 16 Tor Vergata University Hospital, Medical Genetics Unit, PTV, 00133 Rome, Italy; l.b.salehi@mclink.it; 17 Medical Genetics Unit, University of Perugia Hospital SM della Misericordia, 06129 Perugia, Italy; paolo.prontera@ospedale.perugia.it; 18 Department of Pediatrics, Clinical Genetics, Università di Padova, 35122 Padova, Italy; romano.tenconi@unipd.it; 19 Department of Brain and Behavioral Sciences, University of Pavia, 27100 Pavia, Italy; luigi.corsaro01@universitadipavia.it

**Keywords:** oculo-auriculo-vertebral spectrum, OAVS, DNA-methylation, genome-wide, infinium human methylation 450K beadchip, retinoic acid

## Abstract

Oculo-auriculo-vertebral-spectrum (OAVS; OMIM 164210) is a rare disorder originating from abnormal development of the first and second branchial arch. The clinical phenotype is extremely heterogeneous with ear anomalies, hemifacial microsomia, ocular defects, and vertebral malformations being the main features. *MYT1*, *AMIGO2*, and *ZYG11B* gene variants were reported in a few OAVS patients, but the etiology remains largely unknown. A multifactorial origin has been proposed, including the involvement of environmental and epigenetic mechanisms. To identify the epigenetic mechanisms contributing to OAVS, we evaluated the DNA-methylation profiles of 41 OAVS unrelated affected individuals by using a genome-wide microarray-based methylation approach. The analysis was first carried out comparing OAVS patients with controls at the group level. It revealed a moderate epigenetic variation in a large number of genes implicated in basic chromatin dynamics such as DNA packaging and protein-DNA organization. The alternative analysis in individual profiles based on the searching for Stochastic Epigenetic Variants (SEV) identified an increased number of SEVs in OAVS patients compared to controls. Although no recurrent deregulated enriched regions were found, isolated patients harboring suggestive epigenetic deregulations were identified. The recognition of a different DNA methylation pattern in the OAVS cohort and the identification of isolated patients with suggestive epigenetic variations provide consistent evidence for the contribution of epigenetic mechanisms to the etiology of this complex and heterogeneous disorder.

## 1. Introduction

Oculo-auriculo-vertebral spectrum (OAVS; OMIM 164210) is a rare craniofacial disorder involving structures derived from the first and second branchial arches with an estimated prevalence of 3.8 per 100,000 births [1]. The OAVS phenotype is clinically heterogeneous and mainly characterized by hemifacial microsomia, bilateral or unilateral dysplastic ears (anotia, microtia, anomalies of the pinnae, preauricular pits, and tags), hearing loss (conductive or sensorineural), ocular defects (epibulbar dermoids, microphthalmia, coloboma of upper eyelid), and vertebral malformations. Cleft lip or palate, cardiac, renal, cerebral malformations, and intellectual disability were also described as additional features [1,2,3,4,5]. Minimal diagnostic criteria suggested by several authors include isolated microtia or hemifacial microsomia, together with mild ear malformations, such as preauricular tags and pits [6,7].

The etiology of OAVS is largely complex and heterogeneous. Environmental factors such as maternal diabetes, retinoic acid, or vasoactive drug exposure during the pregnancies, and bleeding of placenta vessels have been proposed to be causative of this condition [8,9,10]. A genetic contribution has been demonstrated by the description of autosomal dominant and recessive families [11,12,13], the detection by linkage analysis of locus on chromosome 14q23, including the Goosecoid (*GCS*) gene, the screening of which has resulted negative [12,14], and the identification of several chromosomal abnormalities, including recurrent anomalies on chromosomes 5p, 14q, and 22q [3,6,8,15,16,17,18,19,20,21]. Recently, variations in a few genes have been described in OAVS patients, including *de novo* changes in the myelin transcription factor 1 (*MYT1*) gene, involved in the retinoic acid metabolism, [22,23] and in the *AMIGO2* and *ZYG11B* genes, the former participating to the (PI3K)/AKT signaling pathway [24], and the latter involved in proteasome degradation [25].

The identification of discordant monozygotic twins and the high frequency of OAVS children born by assisted reproduction technologies suggest that epigenetic mechanisms may also contribute to the etiology of OAVS [26,27,28]. Consistent with this hypothesis is the identification in OAVS patients of an allelic expression imbalance dependent on histone acetylation in the *BAPX1* gene that can be successfully rescued by treating cells with histone deacetylase inhibitors [29]. *BAPX1* encodes for a member of the NKX family of homeobox-containing transcription factors and plays a role in both skeletal development and patterning of the middle ear [29,30,31]. These observations are corroborated by studies on animal models that allowed the identification of further histone deacetylases involved in the epigenetic control of skull morphogenesis, [32].

Epigenetic modifications are generally defined as the result of processes that lead to inheritance not related to the nuclear DNA sequence. Basically, these mechanisms are divided into two categories which include modifications of histones and modifications of DNA itself by methylation of CpG nucleotides [33].In particular, the DNA methylation process regulates gene expression by recruiting proteins involved in gene repression or by inhibiting the binding of transcription factors to DNA. However, the process of regulating gene transcription is a complex mechanism in which several epigenetic mechanisms participate. To regulate transcription, DNA methylation interacts with histone and microRNA modifications [34].

Considering the crosstalk of DNA methylation and other epigenetic mechanisms, and the data supporting the possible involvement of histone modifications in the etiology of OAVS, we hypothesized that an alternative/complementary mechanism of gene expression regulation such as DNA methylation could contribute to the disease. To this aim, we used a genome-wide approach to compare the DNA-methylation profile of 41 OAVS patients and 48 ethnically-matched controls. Array-based genome-wide DNA methylation analysis provides an unprecedented opportunity to evaluate the DNA methylation contribution to OAVS etiology, which has not yet been investigated.

## 2. Results

### 2.1. OAVS Patients and Controls

We took advantage of Infinium Methylation 450K Beadchip technology to compare the methylation profile of an OAVS cohort (*n* = 41) to that of a group of selected healthy controls (*n* = 48). The main clinical features found in our OAVS cohort are summarized in Table 1, while the detailed clinical information of each of the 41 unrelated OAVS patients is provided as Supplementary Table S1. Minimal inclusion criteria were a suggestive clinical diagnosis of OAVS consistent with the commonly used criteria (e.g., ear anomalies and hemifacial microsomia) [6,7]. The OAVS group was constituted of 19 females and 22 males, with a median age of 6 years. As summarized in Table 1, craniofacial anomalies (e.g., hemifacial microsomia and mandibular hypoplasia) and ear anomalies characterized almost all patients. The prevalence of these OAVS main features in our cohort was concordant with the prevalence in OAVS patients from literature data [1,6,7]. The control group included 48 tissue-matched methylation profiles of individuals referred to as not presenting relevant or suggestive signs of OAVS or other syndromes (age median: 12.9 years).

### 2.2. Group Level: Differential Methylation Analysis

**Figure 1:**
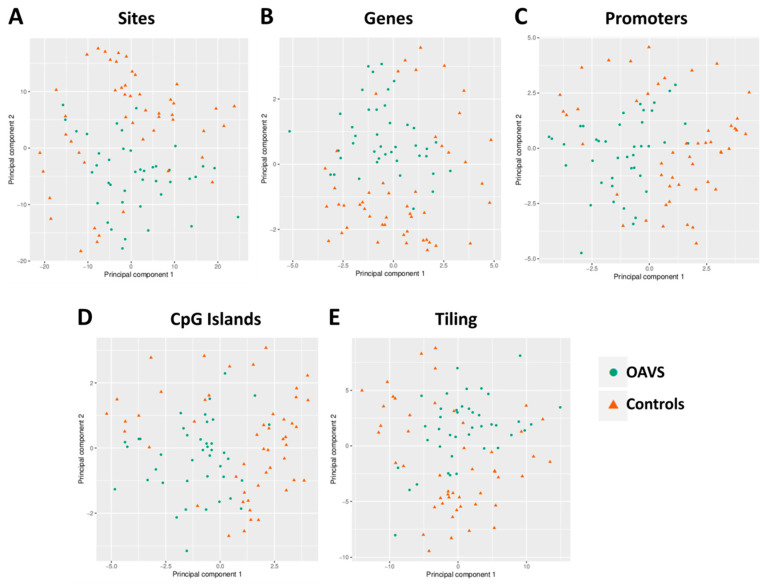
Scatter plot locating samples in the first two principal components at (**A**) sites, (**B**) genes, (**C**) promoters, (**D**) CpG islands, and (**E**) tiling.

We used an integrated and custom pipeline to identify the methylation differences at the group and at the single case levels. Group analyses were mainly carried out using the specific modules of the RnBeads package. Principal component analysis (PCA) was considered to reduce data complexity and evaluate variations in the methylation profiles of five selected genomic regions such as sites, genes, promoters, CpG islands, and tiling. Analysis did not reveal any strong variation neither among patients nor between patients and controls, since no separation was observed along the first and the second components in all considered regions (variance explained: Sites: PC1 = 17.9%, PC2 = 13.8%; Genes: PC1 = 22.4%, PC2 = 14.2%; Promoters: PC1 = 20.9%, PC2 = 18.51%; CpG Islands: PC1 = 28.7%, PC2 = 9.34%; Tiling: PC1 = 24%, PC2 = 11.2%) (Figure 1).

Moreover, PCA analysis carried out also on additional regions such as imprinted-associated Differentially Methylated Regions (DMR) and Retinoic Acid Response Elements ((RARE) ± 10 Kb upstream/downstream of selected RAREs—coordinates available in Supplementary Table S2) did not find any striking split between OAVS and controls cohorts (Supplementary Figure S1).

**Figure 2:**
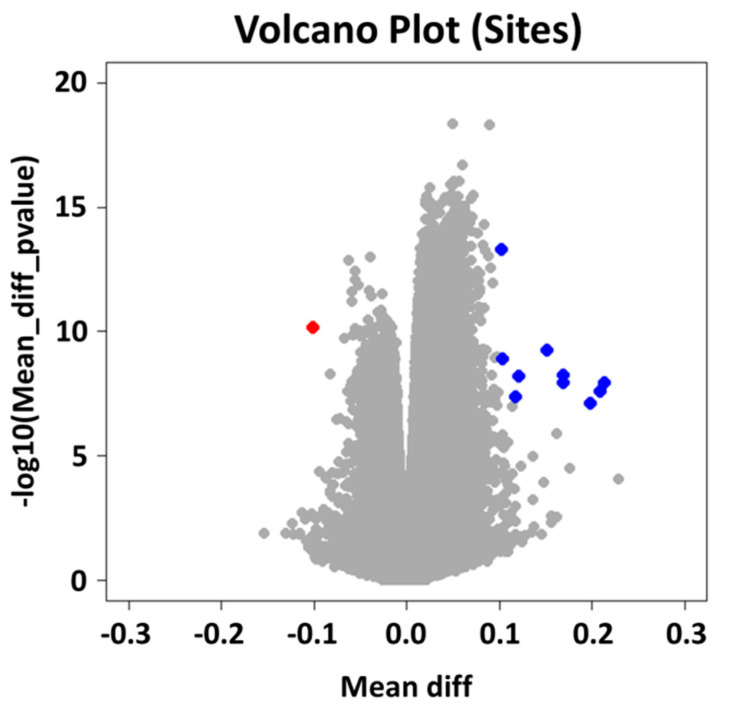
Volcano plot for differential methylation: CpG sites meeting *p*-value and methylation difference thresholds are depicted as red (hypo-methylated) and blue (hyper-methylated) dots.

The absence of polarization of OAVS samples from controls was also confirmed by the unsupervised hierarchical clustering based on 1000 most variable loci showing patients and controls randomly mixed (Supplementary Figure S2). Besides, we did not identify any differences in epigenetic age and cellular components of peripheral blood between the two cohorts (OAVS vs. controls) (Supplementary Figure S3). To identify differentially methylated sites and regions (Genes, Promoters, CpG Islands and Tiling regions) between sample groups, we used the integrated Differential Methylation module of RnBeads, enabling surrogate variables analysis (SVA) to correct for systematic confounders. At the site level, the analysis highlighted 46,017 differentially methylated positions with a genome-wide threshold (*p* < 10^−7^) (the complete list of significant differentially methylated CpG sites is provided in Supplementary Table S3). To decrease the number of significant CpGs and identify sites with more biologically relevant methylation differences we applied an additional cutoff to the methylation differences between the two groups (diff meth > |10%|): the number of significant sites decreased to 11 sites, 10 hyper-methylated and 1 hypo-methylated, as depicted in the Volcano plot (Figure 2—red and blue dots), confirming a predominance of significant but very slight epigenetic changes. The last filtered probes were too few to try any enrichment strategy.

We then focused our attention on the differential analysis performed at the region level (genes, promoters, CpG Islands, and tiling). We obtained, for each region type, thousands of positions with significant (padj < 0.05) but very small differences between OAVS and controls (Supplementary Tables S4–S7). To strengthen and prioritize our results, we applied (i) a more stringent cutoff for *p*-values adjusted for multiple tests (padj < 0.01), (ii) for each region a minimal threshold to the differences in methylation means (diff meth>|10%|) and (iii) at least 2 sites associated with the regions. After the additional filtering steps, we were not able to identify any significant differentially methylated regions. Conversely, concerning CpG islands and tiling, we identified one hyper-methylated position (at CpG islands and tiling levels) encompassing an intronic or exonic region of the *PPFIA4* gene (chr1:203,040,001–203,045,000 (hg19). To further dissect and explore our results, we linked the large differentially methylated gene lists to biological functions by performing a gene ontology (GO) enrichment analysis through the relative RnBeads module. The top 50 significantly enriched-GO terms (biological processes) from the best 100 ranked hyper- and hypo-methylated genes are reported as Supplementary Table S8. To summarize and classify GO terms into macro-categories, we used ReviGO (Reduce+Visualize Gene Ontology) tool to cluster similar GO terms as tree-maps [35]. Results are depicted as Gene Ontology tree-maps (Supplementary Figure S4). Clustering semantically divided GO-terms enriched in hyper-methylated gene regions into a few main categories such as the immune response to cytokines, organization of DNA or chromatin organization, regulation of several metabolic processes (Supplementary Figure S4A). On the contrary, the clustering of hypo-methylated-derived GO-terms revealed a very heterogeneous pattern of biological processes without highlighting any suggestive pathway (Supplementary Figure S4B).

### 2.3. Differential Methylation at Single Case Level: Stochastic Epigenetic Variants (SEV)

**Figure 3:**
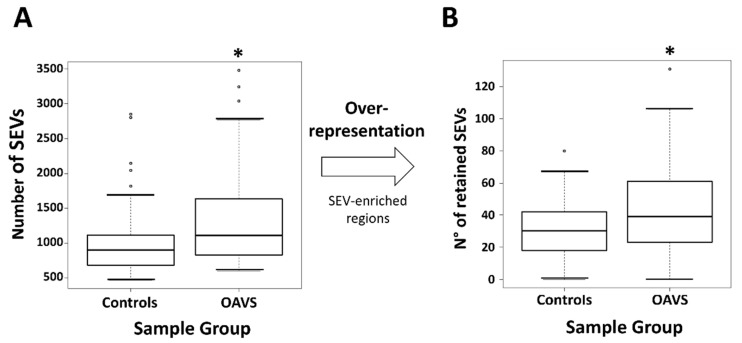
(**A**) Boxplot showing the distribution of SEVs in controls and OAVS patients. (**B**) Boxplot showing the distribution of retained SEVs in controls and OAVS patients after the enrichment analysis. The thick horizontal line represents the median of the distribution while the box represents the interquartile range. Whiskers are set as the default option for the boxplot function and extend to the most extreme data point, which is no more than 1.5 times the interquartile range from the box. Open circles represent outliers (single values exceeding 1.5 interquartile ranges).

The burden of SEVs in the OAVS cohort was calculated as described in the “Methods” section and compared to that observed in controls: the number resulted significantly higher (Figure 3A). To identify significantly SEV-enriched regions, for each patient and each control, we carried out an over-representation analysis by using a sliding window algorithm based on a cumulative hypergeometric test (see Materials and Methods for details). When we compared OAVS and controls for the number of retained CpGs, we observed that this number remains significantly higher in the OAVS group (Figure 3B).

Besides, to increase the specificity of our results, we cleaned the SEV lists of OAVS patients from the deregulations observed in the control group, tendentially considered non-specific or physiological. The lists of contiguous deregulated CpGs were then annotated to genes. Relative gene frequencies and the extent of epigenetic deregulations (n° retained SEVs) of the most shared gene loci are shown in Table 2 (hyper-methylated) and Table 3 (hypo-methylated). Interestingly, although many outlier beta-values were identified in some OAVS patients, the enrichment analysis did not yield any deregulated locus. This is the case of three patients (OAVS07, OAVS36, and OAVS47). The results show a substantial enrichment of univocal hyper-methylated loci compared to hypo-methylated ones (145 vs. 27). Consistent with the results obtained from the groups’ comparison, we did not notice a wide sharing of deregulated gene loci among the OAVS patients: the deregulation involves single samples in most cases. Among the most shared hyper-methylated genes, to note the *PPIAF4* gene, already described above, with 6 cases with 4/5 contiguous SEVs located in the 3′ region of the gene (exon 29) and *ISOC2*, *LLPH*, and *RGMA* genes involving 3 cases each.

Genes enriched in hypo-methylated SEVs are numerically lower than hyper-methylated ones: the most frequent deregulated loci are *UPP1*, *HOXA3*, and *LOC100507547*, involving two cases each.

The full list comprising all deregulated gene loci is provided as Supplementary Tables S9 and S10.

#### Annotation and Prioritization Analysis

**Figure 4:**
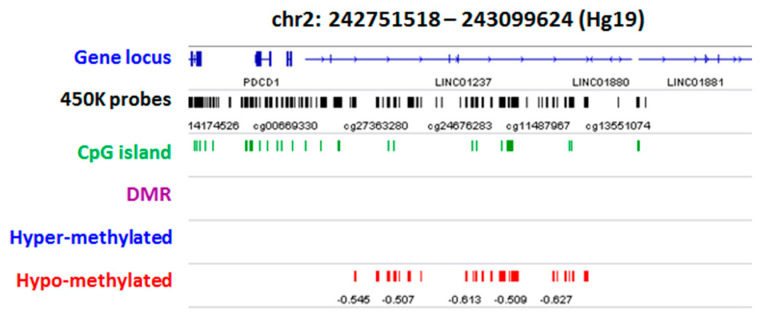
IGV screenshot of the alignment to the genome of the deregulated CpGs of patient OAVS08. The first track shows the map of RefSeq genes. 450K probes and relative CpG Islands are reported in the second and third track, respectively. Hypo-methylated sites are shown in red color.

Prioritization was carried out through the computational tool Phenolyzer (phenotype-based gene analyzer) (http://phenolyzer.wglab.org/) [36]. Analyses returned a list of genes with a low normalized score indicative of a poor association with seed genes linked to selected keywords. However, we found a relatively high association with the “craniofacial” term by combining the keywords. For example, among the most ranked genes enriched in hyper-methylated probes, we discovered genes such as Guanine Nucleotide Binding Protein ((GNAS) G Protein), Alpha Stimulating—Chromosome 20) (8 contiguous hyper-methylated probes (chr20:57465439-57465775) in the promoter of the bi-allelic isoform of GNAS locus) and *EDN3* (Endothelin 3—Chr. 20) (Supplementary Figure S5). On the other side, the prioritization of hypo-methylated enriched genes highlighted genes including *RPS6KA2* (Ribosomal Protein S6 Kinase A2—Chr. 6), Signaling Receptor and Transporter of Retinol ((STRA6) STRA6—Chromosome 15), and *MSX1* (Msh Homeobox 1—Chr. 4) (Supplementary Figure S6). We then performed a literature-based gene prioritization: first, we screened our gene-associated deregulations by intersecting the gene lists with those confirmed or candidates to be causative of OAVS that emerged from both genetic (exome sequencing) or cytogenetic (SNP-array) studies. No epigenetic deregulations were found to be associated to *MYT1* gene [22,23] or others proposed loci including *ZYG11B* [25], *AMIGO2* [24], *OTX2*/*YPEL1*/*CRKL* [21,37], and *NKX3-2* (*BAPX1*) [29]. The only positive matching was found for the gene Xyloside Xylosyltransferase1 (*XXYLT1*) localized in a region previously reported for a de novo microduplication on chromosome 3 (3q29) in an OAVS patient [38]. This gene is enriched in hypo-methylated CpGs in the 3’ terminal region of the gene in one patient (OAVS05). No epigenetic deregulations were also found by inspecting our deregulation lists for the presence of genes causing other syndromes in differential diagnosis with OAVS such as *TCOF1, POLR1C, POLR1D* (Treacher–Collins) [39,40], *CHD7* and *SEMA3E* (Charge) [41,42], *SALL1* (Townes–Brocks) [43] and *EYA1*, *SIX1*, and *SIX5* (Branchiootic syndrome 1) [44,45]. We also searched for deregulated enrichment in imprinted regions (http://www.imprinting-disorders.eu/). The comparison yielded two OAVS samples with distinct deregulation associated with imprinted regions. Sample OAVS4 showed five out of seven probes hypo-methylated in the germline maternally methylated *ERLIN2* gene (intron 6; chr8:37604992-37606088), while sample OAVS29 had six out of ten probes hyper-methylated in the secondary paternally methylated *ZNF597:TSS-DMR* (chr16:492828-3494463). The probes identifiers and relative coordinates are supplied as Supplementary Table S11. The SEV lists were also visually inspected using a Genome Viewer (Integrative Genomics Viewer (IGV)) to detect other possible candidate regions. This approach highlighted a sample (OAVS08) with a consistent hypo-methylation (10 CpG sites) in the telomeric region of chromosome 2. Deregulation has become more evident (78 hypo-methylated probes) by detecting SEVs from beta matrices obtained from RnBeads [46], which, despite ChAMP [47], does not massively filter the proximal telomeric regions in the quality control phase (Figure 4).

## 3. Discussion

In this study, we report a genome-wide methylation analysis of a cohort of 41 OAVS patients by using a 450K Bead Chip array. The differential methylation analysis at the group level highlighted numerous but small significant deregulations involving thousands of regions and genes (Supplementary Tables S3–S6). By applying a Gene Ontology enrichment we obtained a very heterogeneous pattern of GO terms enriched in both hyper- and hypo-methylated genes without emphasizing any suggestive pathway or GO term cluster. However, we observed the deregulation of numerous biological processes involving basic chromatin dynamics such as DNA packaging, chromatin assembly, protein-DNA organization, and gene silencing (Supplementary Table S8; Supplementary Figure S4). In this regard, it is interesting the identification of the hypo-methylation of *ARID1B*, a key component of SWI/SNF chromatin remodeling complexes, as well as the hyper-methylation of several Histone 1 components. A possible involvement of mechanisms regulating chromatin structure in OAVS would be consistent with the previous observation of the histone acetylation dependent allelic expression imbalance of *BAPX1* in OAVS patients [29]. Present and current findings seem to favor more a model in which chromatin structure, rather than DNA methylation, is the epigenetic mechanism playing the major role in OAVS. Nevertheless, epigenetic regulation is a very complex mechanism, which involves, among all, histone modifications and DNA methylation. It is now established that all these changes interact with each other (and influencing each other) and, to date, a clear picture about the causality of events does not yet exist. For example, it cannot be excluded that the weak DNA methylation alterations observed in OAVS patients have consequences on chromatin structure. Collection of further evidences is mandatory to determine whether one of these deregulated processes or the combination of all impact the OAVS pathology.

In the present study, using an approach based on a non-parametric test [48,49], we searched for CpG sites with extreme aberrant methylation status (namely SEVs) in single OAVS patients and noticed a significant increase in the burden of SEVs in OAVS that was maintained even after the enrichment analysis step. These findings support the hypothesis that OAVS patients improperly accumulate SEVs, which have been recently defined as a potential measure of a potential exposure-related accumulation of DNA damage [50,51]. The annotation of the SEV-enriched regions identified 172 deregulated regions, including 145 hyper-methylated and 27 hypo-methylated loci. The absence of highly shared deregulations strengthens the hypothesis of a broad epigenetic heterogeneity in OAVS, though some hyper- and hypo-methylated regions were found in more than a sample. Focusing on the most frequently deregulated loci, we observed that six samples had hyper-methylation of the *PPFIA4* gene (Table 2). This gene encodes a KIF1A-binding protein, which is involved in the trafficking of LAR subfamily of protein-tyrosine phosphatase and AMPA-type glutamate receptors. *PPFIA4* is expressed in the heart, brain, and skeletal muscle [52]. Epigenetic deregulation is contained in a CpG Island localized in the exon 29 of the gene. According to study inclusion criteria, patients with *PPFIA4* hyper-methylation had mandibular hypoplasia and earing defects or preauricular pits and tags, whereas other OAVS-related signs like cardiovascular and vertebral anomalies were variably represented. Interestingly, few OAVS patients displayed the hyper-methylation of the region proximal to the promoter of three genes, namely *ISOC2*, *LLPH*, and *RGMA*. Given the position of the deregulation, we can speculate about a repressed state of the associated genes. Information is only available for the *RGMA* gene. The *RGMA* gene encodes for a member of the repulsive guidance molecule family, which is involved in the regulation of axon guidance in the adult central nervous system by binding the type 1 transmembrane protein Neogenin. This protein also serves as a co-receptor for bone morphogenetic proteins (BMPs) to regulate iron metabolism, skeletal development, axon regeneration, and angiogenesis [53]. Among the hypo-methylated loci, *HOXA3*, *UPP1*, and *LOC100507547* should be mentioned, which were found to be hypo-methylated in two samples each. *HOXA3* was hypo-methylated in an intronic CpG island. This gene encodes for a homeobox protein expressed in the cranial neural crest cells that contribute to the formation of the third and fourth pharyngeal arch and the endoderm of the pharyngeal pouches [54]. The following evidence underlines the relevance of this gene to craniofacial anomalies: (i) Hoxa3 homozygous null mutant mice die shortly after birth and exhibit a wide range of abnormalities in the neck and craniofacial structures [55,56,57]; (ii) *HOXA3* expression is regulated by the retinoic acid (RA) during embryonic development: alteration of the expression of RA and consequent *HOXA3* gene is reported to be a cause of craniofacial and thymus anomalies [58,59] and more recently heart defects [60,61]; (iii) *MYT1*, the first gene that was clearly associated with OAVS, is also involved in the RA pathway [22,23]. In addition to *HOXA3*, two other genes involved in neural crest differentiation and the RA pathway showed enrichment of hypo-methylated sites: *MSX1* and *STRA6*. Interestingly, the hypo-methylation of *STRA6* was described in a patient showing also altered methylation of *RGMA* and *HOXA3*. *MSX1* is a transcriptional factor that is highly expressed during embryogenesis and postnatal development in the bone. It acts as a transcriptional repressor crucial in palatogenesis and odontogenesis but is also involved in limb anomalies and cleft lip or palate malformations [62]. Inactivating variants of the *MSX1* gene have been reported to be causative of craniofacial anomalies in rare congenital diseases such as tooth-and-nail (Witkop) or Wolf–Hirschhorn syndromes [63]. In a mouse model, the lack of Msx1 function is causative of the Pierre–Robin Sequence, which manifests with important oro-facial anomalies [64]. Our patient showed multiple hypo-methylated CpG sites in a CpG island encompassing the second exon of the *MSX1* gene, which encodes for the gene homeodomain (HD). The HD is essential for protein stability, DNA binding, transcriptional repression, and interactions with other odontogenic molecules like *PAX9*, TATA-binding protein (TBP), and DLX family members [63]. The hypo-methylation of CpG sites located in the exon 2 of the *MSX1* has been described in epithelial ovarian cancer and causes a decrease in gene expression, whereas point mutations affecting the *MSX1* HD cause tooth agenesis with or without other phenotypes [56]. Of note, also our OAVS patient with *MSX1* hypo-methylation showed a dental phenotype consisting of small teeth with multiple caries. Besides, craniofacial anomalies, multiple auricular tags, oesophageal atresia, alternate strabismus, vertebral anomalies, and radial defects at the right side, including absent radius, ulnar hypoplasia, and hypoplastic thumbs were also present. Moreover, we cannot exclude that a decreased expression of this gene may influence the correct development of other body structures altered in OAVS since *MSX1* regulates the activity of other genes [65]. The hypo-methylation of the first exon of the *STRA6* gene was identified in a patient with full-blown OAVS phenotype, including mandibular and ear anomalies, epibulbar dermoid, and cardiovascular defects. This patient had concomitant hypo-methylation of the *RGMA* and *HOXA3* genes. Remarkably, *STRA6* encodes a trans-membrane retinol transporter, which mediates cellular uptake of vitamin A and is transcriptionally up-regulated by RA exposure [66] that prenatally retinoic acid (RA) exposure has been associated with craniofacial anomalies. Remarkably, mutations in the *STRA6* gene are seen in ocular defects in humans, such as coloboma and microphthalmia [67], and our patient presented eye coloboma, suggesting a potential and specific contribution of the hypo-methylation to the eye phenotype of the patient.

Among the genes that emerged from prioritization analysis with Phenolyzer, we also report the involvement of *GNAS* locus in a single patient. The epigenetic deregulation (8 CpG sites) was located in a CpG island closed to *GNAS* A/B:TSS-DMR and encompassed the promoter of the biallelically expressed alpha subunit of the stimulatory G protein, which plays essential roles in a multitude of physiologic processes [68]. Inactivating loss-of-function mutations in *GNAS* cause pseudohypoparathyroidism type 1a (*PHP1A*), pseudopseudohypoparathyroidism, and progressive osseous heteroplasia [69]. Importantly, recent findings associate *GNAS* mutations specifically to the particular craniofacial alterations and dental abnormalities found in some *PHP1A* patients [70].

As reported in the results section, we also searched for epigenetic deregulations in genes previously involved in OAVS, assuming that potentially epimutations could mimic the effect of gene and genomic variants. Except for the *XXYLT1* locus, we did not find any positive correspondence. The epigenetic deregulation (hypo-methylation) in *XXYLT1* is confined to the 3′ terminal region of the gene and was described in a single patient. This gene is included in a previously reported microduplication of 723 Kb on chromosome 3q29 in an OAVS patient with mandibular hypoplasia, preauricular pits and tacìgs, hearing loss, coloboma, microphthalmia, cardiac anomalies, and mild intellectual disability [38]. *XXYLT1* encodes an endoplasmic reticulum localized xylosyltransferase that regulates the activation of *NOTCH1* by adding xylose to the Notch extracellular domain. An animal model has shown that Notch signaling plays a primary role in establishing left-right asymmetry in mice by directly regulating the expression of the Nodal gene [71].

The analysis of imprinted regions highlighted two positive matches, a hypo-methylation of the germline maternally methylated *ERLIN2* and a hyper-methylation of the secondary paternally methylated *ZNF597*: TSS-DMR region. To date, incomplete or no information is available about these two differentially methylated regions; therefore, we could not explain if these deregulations may be associated with the disease. In addition, we found a large demethylated region on chromosome 2 in a subject with hemifacial asymmetry, mandibular hypoplasia, preauricular tags, hearing loss, monolateral dysplastic ear, dorsal vertebral malformations, ventricular septal defect, pyelectasis, polycystic kidney, anal atresia, and curved penis. SNP-array analysis performed on this patient indicated the presence of a homozygous deletion, representing a common variant reported on the DGV database (http://dgv.tcag.ca/dgv/app/home). However, it is not possible to deduce from the database whether the deletion can be considered a benign variant in a homozygous state as well, and therefore we cannot exclude that this demethylated region contributed to the patient’s phenotype.

We are aware of the limitations that should be considered when analyzing and interpreting genome-wide methylation results. We choose whole blood as representative of the methylation status of the disease: complementary studies on additional tissues should be carried out to confirm results. A larger sample size would be preferable to better estimate normal and pathological ranges of DNA methylation for each CpG site. Our study analyzed and evaluated mainly rare epigenetic alterations, never identified in controls but shared in a very small number of patients. Another limitation of the present study is that we did not evaluate the impact of the epigenetic variants at the transcriptional level, an investigation necessary to define the potential role of these rare deregulations in OAVS etiology.

In conclusion, this study represents the first epigenetic investigation on DNA methylation in OAVS patients and confirms the high complexity of this disease. Both group- and single-case analyses support the epigenetic heterogeneity of the disease, identifying in few patients some suggestive and consistent deregulations, which mainly affect genes involved in cranial and facial development, such as in *HOXA3*, *MSX1*, and *STRA6*, whose expression is modulated by retinoic acid. An increasing understanding of these potentially causative mechanisms may contribute to an accurate dissection of this heterogeneous disease and, therefore, to a more accurate diagnosis for the patients and possible medical treatments, including a personalized follow-up, from counseling to reconstructive and rehabilitative steps due to maxillofacial and skeletal anomalies.

## 4. Materials and Methods

### 4.1. Study Design and Population

The study cohort included 41 OAVS patients comprising 38 sporadic cases and 3 individuals with family history, belonging to 41 independent families. Patients were aged from 5 days to 46 years and included 19 females (46%) and 22 males (54%). The great majority of the patients were of Caucasian origin, with only 6 patients from Brazil (OAVS42), Bangladesh (OAVS20), Sri-Lanka (OAVS16), Colombia (OAVS44), India (OAVS29), and Santo Domingo (OAVS18), respectively. Clinical data were collected using a protocol shared by all the clinicians participating in the study, including the following clinical information: family history, reproductive and pregnancy history, clinical and phenotypic genetic evaluation. Except for two samples (OAVS31 and OAVS02), all patients underwent *MYT1* sequencing in the coding region and intron-exon boundaries, and no pathogenetic variant was identified. This study was approved by the Institutional ethical committee of Casa Sollievo della Sofferenza Hospital, San Giovanni Rotondo 13 November 2009. Written informed consent was obtained from all patients. The reference population has been selected according to recommendations suggested by the most important guidelines. Since age represents the most important source of biological variability in our study, we selected, as best as possible, age-matched subjects and checked that cases and controls were not significantly different in terms of age. The final control cohort was composed of 48 tissue-matched methylation profiles of anonymous healthy individuals processed in the same batch or resulting from unrelated parallel studies (age median: 12.9 years; 34 females and 14 males).

### 4.2. DNA Extraction

Genomic DNA was extracted from peripheral blood lymphocytes using a manual kit, according to manufacturer instructions (Mackerey–Nagel). Quality control and quantification of DNA was assessed by visualization of genomic DNA (gDNA) on 1% agarose gel electrophoresis and by using NanoPhotometer Pearl (Implen GmbH).

### 4.3. Bisulfite Conversion

In total, 800 ng of gDNA were-bisulfite converted by using the EZ DNA Methylation Kit (Ref: D5001, Zymo Research Corporation) according to the manufacturer’s protocol. Specific incubation conditions (Illumina Protocol) were applied. To evaluate conversion efficiency and bsDNA integrity, a single-strand quantification of bisulfite converted DNA (bsDNA) was performed by using NanoPhotometer Pearl (Implen GmbH). Fragmented or too diluted DNA samples were discarded and then reprocessed.

### 4.4. Genome-Wide Methylation Analysis

**Figure A1:**
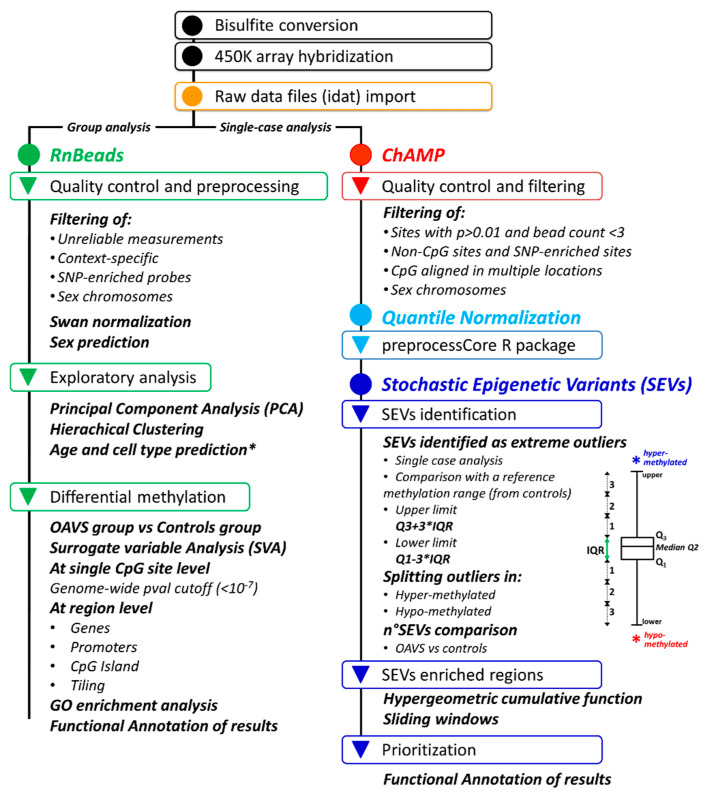
Schematic representation of the multistep analysis pipeline. Genomic DNA was subjected to bisulfite conversion and used for the methylation array procedure. The analysis pipeline has been structured to follow two different but complementary approaches: one (left side) aimed at group comparison by using the integrated functions of the RnBeads package in the R environment. The second one (right side) was structured to analyze single cases through the development of a custom multistep cascade involving both available packages (ChAMP) and homemade scripts. Analyses are described in detail in the methods sections.

A 450K array-based procedure was carried out following the manufacturer’s instructions and using Illumina-supplied reagents and conditions. The genome-wide methylation analysis pipeline used in this study is represented in Figure A1. To combine both group-level and individual-level analyses, we developed two parallel approaches. The first one (Figure A1—left side) was aimed at a group analysis by comparing the methylation profile of the OAVS group vs. that of the reference control group. The second (Figure A1—right side) was set to analyze individual cases by matching, for each CpG site, the single methylation profiles to a reference methylation range (obtained from controls) to detect extreme methylation differences (SEVs) [48,49]. Raw intensities were loaded from idat files by using specific R packages. The methylation value for each CpG site was represented as beta-value. The collection, handling, and processing of the reference samples were performed applying a standardized approach, consistent with the methods used for testing cases. Since batch effect represents the most important potential source of variability, we avoided using external datasets obtained from public repositories as a reference group and analyzed the control subjects in the same experimental condition of cases. We performed normalization and accurate quality control steps: finally, we evaluated methylation data through PCA and confirmed that both cases and controls were comparable.

#### 4.4.1. Differential Methylation Analysis at the Group Level

For the differential methylation analysis conducted at the group level, we used the RnBeads package (2.4.0) [46] in R environment (version 3.6.1): by using its integrated modules, it represents a powerful tool to perform quality control, normalization, and exploratory (e.g., Principal Component Analysis) analyses of raw data as well as differential methylation analysis on different regions (genes, promoters, CpG islands, and tiling regions), or ontology enrichment of differentially methylated genes (Figure A1—left side). SNP-enriched probes (11,036 sites), unreliable measurements (2493 sites), context-specific (3102 sites), and on sex chromosomes probes (11,041 sites) were filtered out in the pre-processing step. As a final outcome of the filtering procedures, 458,815 CpG sites were retained and all samples met the minimal criteria for being included in the study. Signal intensities were normalized using the SWAN normalization method (minfi package) [72]. Differential methylation analysis was conducted according to the sample groups by computing *p*-values using the limma method for the site level analysis. For the analysis of predefined regions (Genes, promoters, CpG island, tiling), a combined *p*-value was calculated from the *p*-values of single sites. The four sets of genomic regions are defined as follows: (i) genes: Ensembl format genes, version Ensembl Genes 75 (*n* = 29,741), (ii) Promoters regions: the regions 1.5 kb upstream and 0.5 kb downstream of the transcription start sites (*n* = 29,918), (iii) CpG Islands: CpG island track of the UCSC Genome Browser (*n* = 25,822), and (iv) Tiling regions: non-overlapping tiling regions with a fixed window size of 5 kilobases defined over the whole genome (*n* = 131,131). We customized RnBeads analysis by defining additional region annotations such as (i) 49 imprinted-associated Differentially Methylated Regions (DMR) (http://www.imprinting-disorders.eu/) and (ii) 3429 regions encompassing a literature-selected list of Retinoic Acid Response Elements (RARE) (±10 Kb) [73]. The relative coordinates are available in Supplementary Table S2. Surrogate Variable Analysis (SVA) was applied in the differential methylation step, by using the function directly provided in the package, to estimate surrogate variables that can account for cell-type composition as well as any other sources of systematic variation or confounders [74]. Prioritization of differentially methylated genes was conducted by GO Enrichment Analysis via RnBeads by using an algorithm (GOstats) based on a hypergeometric test and the hierarchical structure of the gene ontology database [75].

#### 4.4.2. Stochastic Epigenetic Variants (SEVs)

**Figure A2:**
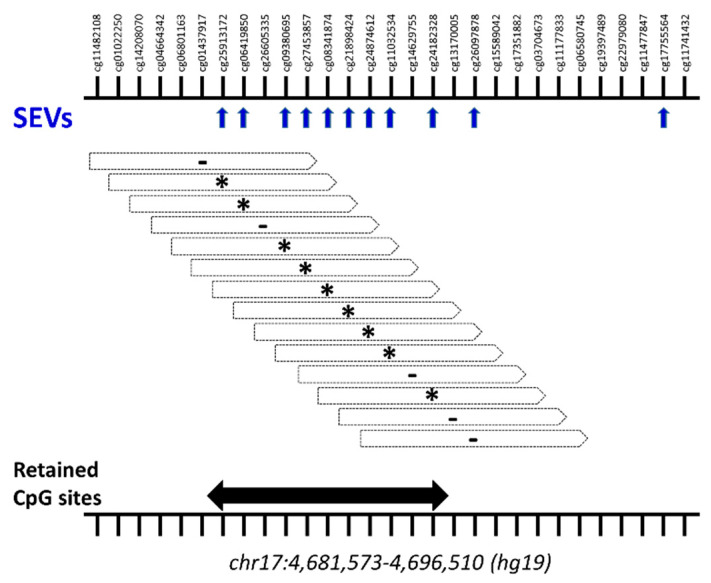
Schematic representation of the functioning of the sliding window approach on an exemplificative OAVS sample by considering an 11 sites window. Arrows show the genomic position of SEVs. Asterisks indicate significant SEV-enriched-windows while, on the contrary, the dashes point to those not meeting the required criteria. The genomic representation of CpG sites is not in scale.

To identify the epigenetic differences in OAVS patients, we adopted a complementary methylation analysis strategy, previously described by Gentilini et al. [48,49] aimed at single case evaluation (Figure A1—right side). Quality control, pre-processing, and generation of the β-values matrix were performed using the ChAMP R package (Chip Analysis Methylation Pipeline—release 2.8.9) [47]. Sites with a detection *p*-value above 0.01 (*n* = 4706) and a bead count <3 in at least 5% of samples (*n* = 226) were removed. Moreover, also non-GpG probes (*n* = 3025), potentially SNP affected probes (*n* = 57,699) [76], probes aligning to multiple locations (*n* = 11) [77] and of X and Y chromosomes (*n* = 9705) were filtered out. As a final outcome of the filtering procedures, a total of 410,140 CpG sites were retained. Quantile normalization was performed through the preprocess core package (release 1.38.1). As represented in Figure A1 (right side), Stochastic Epigenetic Variants (SEV) were identified as beta-values (defined extreme outliers) falling outside a reference methylation range obtained by the methylation profiles of healthy controls (*n* = 48) and calculated as follows: upper value = Q3 + (k × IQR); lower value = Q1 − (k × IQR); where Q1 is the first quartile, Q3 the third quartile, Interquartile range (IQR) = Q3 − Q1 and k = 3. For each case, extreme outlier values of single cases were annotated and classified as hyper-methylated or hypo-methylated with respect to the relative probe median values of controls. To detect SEV-enriched regions, for each case, an over-representation analysis of all identified SEVs (no cutoff on methylation differences was applied) was conducted by using a sliding window algorithm based on a cumulative hypergeometric test that slips on the annotated genome, evaluating the enrichment of SEVs in a window of a predefined size (e.g., 11 CpG sites) and generating a window-associated *p*-value. In the case of statistical significance (*p*-value < default threshold), the algorithm retains and annotates the central CpG site in a new list (if the central CpG was previously detected as an outlier), while on the contrary, is discarded (Figure A2). The procedure is continuously repeated for the next adjacent 11 sites of the Illumina 450K manifest. Through this approach, we can filter spurious and isolated SEVs characterized by an unsure biological meaning for each sample. SEVs approach was finally replicated and extended to the group of healthy controls, analyzing each subject as a case. To strengthen the robustness and specificity of our results, we finally (i) subtracted the SEVs enriched-regions of controls from OAVS methylation enriched-profiles (which were then assigned to genes by using wANNOVAR [78] as for C/T genetic variants) and (ii) excluded retained loci with a single epigenetic variant. To prioritize the results, we used the computational tool Phenolyzer (phenotype-based gene analyzer) (http://phenolyzer.wglab.org/) [36] to combine the lists of hyper- and hypo-methylated enriched genes with some disease-associated terms. We selected general keywords, including differential disease names (e.g., Goldenhar, Charge, Treacher Collins, Townes–Brocks) and suggestive phenotypic traits (craniofacial, dysplastic ears, epibulbar dermoid, genitourinary tract malformation, hemifacial hypoplasia, hemifacial microsomia, preauricular pits, preauricular skin tags, vertebral anomalies).

### 4.5. DNA Methylation Age and Cell Types

DNA methylation ages and proportions of T cells (CD8 naive, CD8, CD4 naïve, CD4), NK cells, B cells, monocytes, and granulocytes were estimated using Steve Horvath’s DNA Methylation Calculator (https://dnamage.genetics.ucla.edu/home) [79].

### 4.6. Statistics

To evaluate differences in age and cell-type composition between cases and controls, the “Wilcox.test” function provided in the R package “class” was used. To assess significant differences in the number of SEVs and SEV-enriched regions between OAVS and controls a Generalized Linear Regression (glm) model adjusted for gender and age was applied. Unless otherwise stated, the statistical significance threshold was set to 0.05.

### 4.7. Data Visualization

PCA charts and Volcano plots were produced by “ggplot2” and “graphics” packages in R, respectively. Density plot was produced by using “sm” package. The clustering of GO biological processes was visualized using a tool such as ReviGO (http://revigo.irb.hr/) [35], setting the parameter “Allowed similarity” to Medium (0.7) and referring to the UniProt-to-GO mapping file “goa_uniprot_gcrp.gaf.gz” dated 15 March 2017.

## 5. Data Repository

All data are deposited to GEO (https://www.ncbi.nlm.nih.gov/geo/) with accession number GSE152204. The function adopted to calculate SEVs is published at DOI:10.5281/zenodo.3813234.

## Figures and Tables

**Table 1:** Summary of the main clinical features of the 41 OAVS patients.

**OAVS cohort**	N = 41	
**Sex**	19 females/22 males	
**Age (years)**	Median = 6 (first quartile = 3—third quartile = 14)	
**Main anomalies**		**Our cohort**
**Craniofacial**	Hemifacial microsomia	39/41 (95%)
	Mandibular hypoplasia	37/41 (90%)
	Cleft lip/palate	8/41 (20%)
**Ear**	Microtia/anotia	41/41 (100%)
	Asymmetric ears	
	Atresia of the external auditory canal	
	Dysplastic ears	
**Ocular**	Microphthalmos	24/41 (58%)
	Coloboma upper eyelid	
	Epibulbar dermoid	
	Other eye anomalies	
**Vertebral**	Hemivertebrae	12/41 (29%)
	Fusion	
	Scoliosis	
	Kyphosis	
	Other vertebral anomalies	
**Cardiovascular**	15/41 (37%)	
**Genitourinary**	7/41 (17%)	
**Brain**	7/41 (17%)	
**Developmental delay**	9/41 (22%)	
**Radial defects**	3/41 (7.3%)	
**Other organs/systems**	8/41(19.5%)	

**Table 2:** Most shared hyper-methylated genes.

Locus	Number of Cases	OAVS Code (N° SEVs)
***PPFIA4***	**6**	OAVS02 (5), OAVS08 (5), OAVS12 (5), OAVS23 (4), OAVS41 (4), OAVS45 (4)
***ISOC2***	**3**	OAVS09 (5), OAVS34 (6), OAVS35 (6)
***LLPH***	**3**	OAVS04 (6), OAVS20 (5), OAVS26 (6)
***RGMA***	**3**	OAVS16 (10), OAVS27 (10), OAVS42 (6)
***ADGB;LOC101928661***	**2**	OAVS14 (7), OAVS32 (4)
***CATSPERE***	**2**	OAVS33 (4), OAVS35 (3)
***CCDC71L***	**2**	OAVS14 (2), OAVS30 (2)
***DDX60***	**2**	OAVS01 (6), OAVS19 (4)
***FRG1;FRG2***	**2**	OAVS20 (7), OAVS28 (5)
***GGCT***	**2**	OAVS26 (6), OAVS33 (3)
***LCLAT1***	**2**	OAVS02 (3), OAVS26 (2)
***LINC01166***	**2**	OAVS26 (6), OAVS02 (3)
***LINC01558***	**2**	OAVS30 (5), OAVS32 (4)
***MIR596***	**2**	OAVS28 (6), OAVS03 (2)
***NPY***	**2**	OAVS30 (5), OAVS16 (3)
***OXGR1***	**2**	OAVS40 (4), OAVS43 (3)
***PLSCR1***	**2**	OAVS33 (8), OAVS02 (3)
***RASSF6***	**2**	OAVS12 (3), OAVS24 (3)
***SIX3;SIX2***	**2**	OAVS32 (5), OAVS40 (4)
***TNFRSF9;PARK7***	**2**	OAVS16 (4), OAVS43 (4)
***VMO1***	**2**	OAVS05 (9), OAVS20 (3)
***ZNF814***	**2**	OAVS35 (7), OAVS21 (4)

**Table 3:** Most shared hypo-methylated genes.

Locus	Number of Cases	OAVS Code (N° SEVs)
***UPP1***	**2**	OAVS27 (5), OAVS42 (5)
***HOXA3***	**2**	OAVS16 (4), OAVS27 (4)
***LOC100507547***	**2**	OAVS27 (6), OAVS16 (2)

## Data Availability

The data that support the findings of this study are available from the corresponding authors upon reasonable request.

## Supplementary Materials

Supplementary Materials can be found at https://www.mdpi.com/1422-0067/22/3/1190/s1.

## Abbreviations

OAVS	Oculo-Auriculo-Vertebral-Spectrum
SEV	Stochastic Epigenetic Variant
PCA	Principal Component Analysis
IQR	Interquartile Range
SVA	Surrogate Variable Analysis
