# Supplementary material for: Genome-Wide DNA Methylation Analysis of a Cohort of 41 Patients Affected by Oculo-Auriculo-Vertebral Spectrum (OAVS)
Source: Int J Mol Sci. 2021 Jan 26;22(3):1190. doi: 10.3390/ijms22031190 (PMC7866060; doi:10.3390/ijms22031190)
Supplement: Supplementary file 1 [file ijms-22-01190-s001.zip › ijms-1072464 supplementary done/ijms-1072464_Suppl. Figures S1-S6.pdf]

International Journal of *Molecular Sciences*

MDPI

Supplementary Figure S1

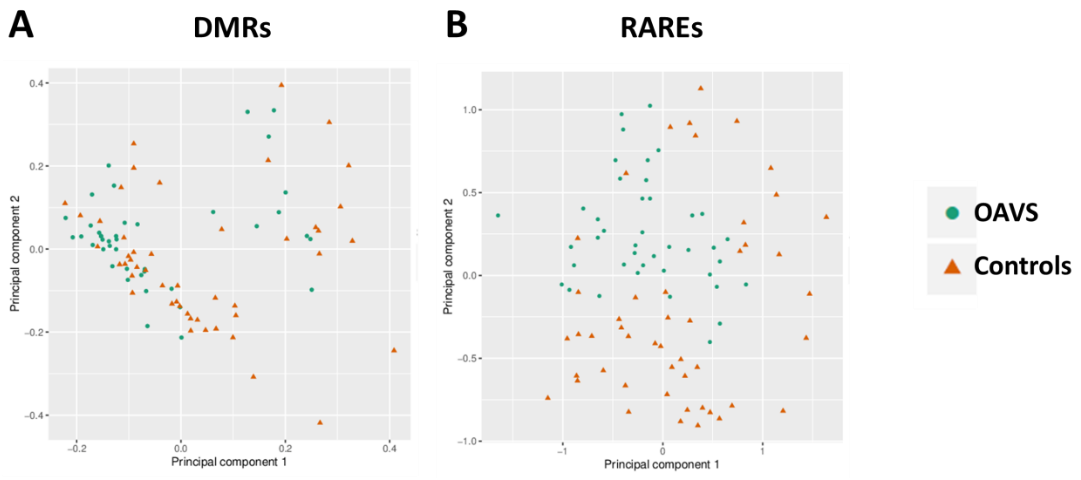

Figure S1. RnBeads scatter plot locating samples in the first two principal components at sites encompassing A) imprinted-associated Differentially Methylated Regions (DMR) (variance explained: PC1=27.44%, PC2=23.20%) and B) Retinoic Acid Response Elements (RAREs) (±10Kb) (variance explained: PC1=24.21%,PC2=15.77%).

Supplementary Figure S2

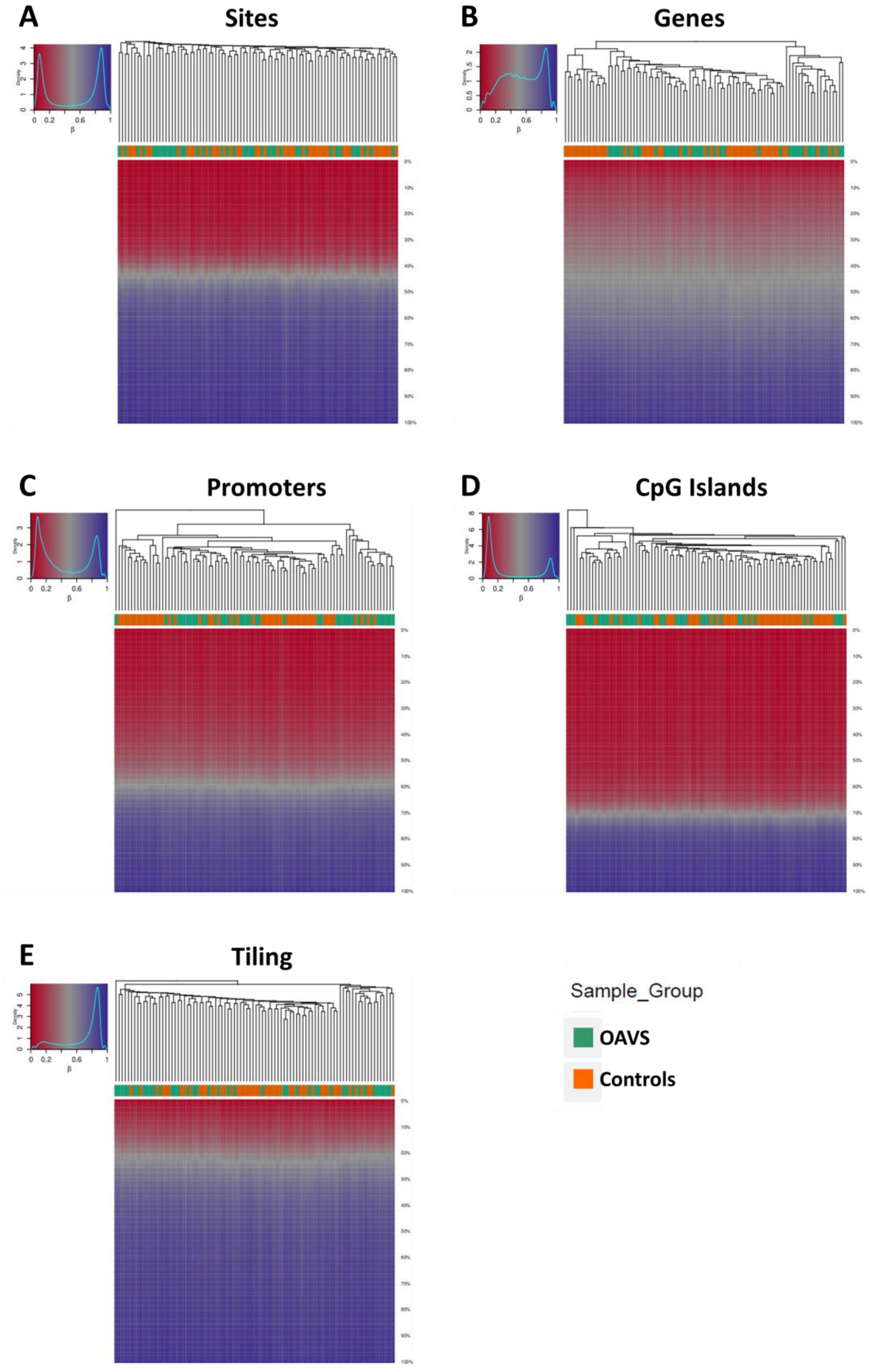

Figure S2. Hierarchical clustering of samples based on 1000 most variable loci. The heatmap displays methylation percentiles per sample (based on beta-values). **(A)** sites, **(B)** genes, **(C)** promoters, **(D)** CpG island, and **(E)** tiling regions. OAVS samples are depicted as orange bars while controls as green.

Supplementary Figure S3

A)

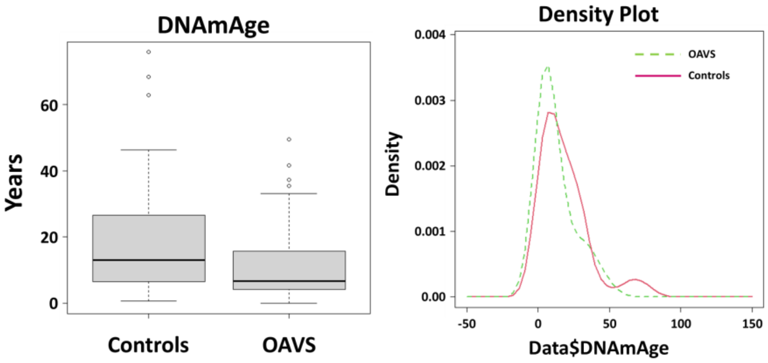

B)

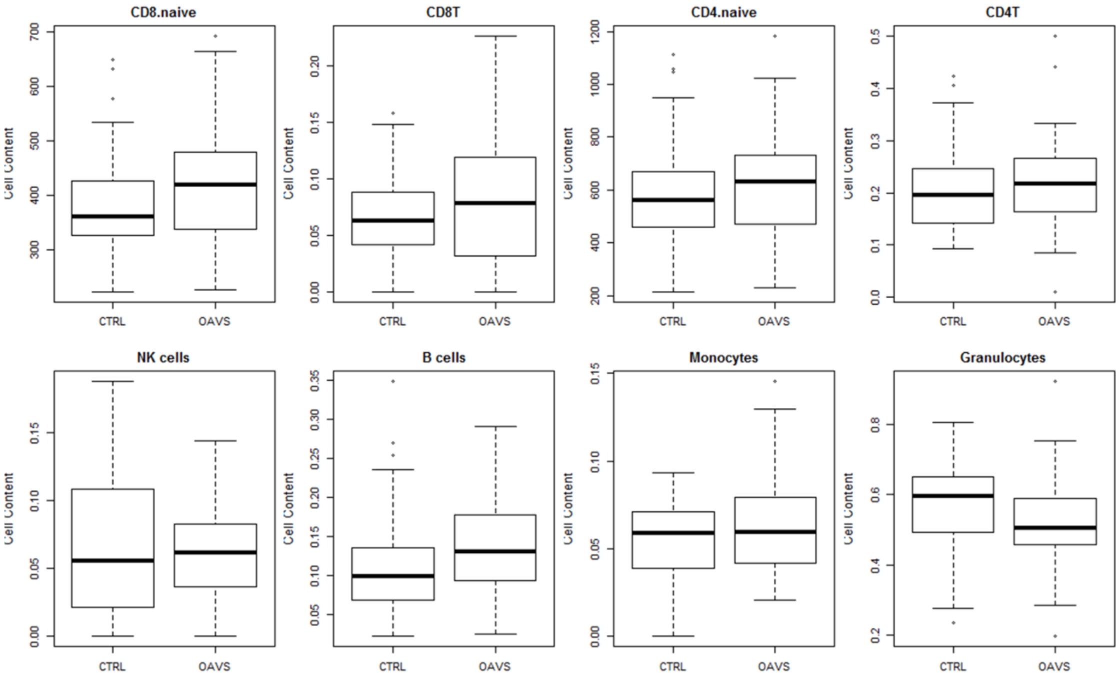

Figure S3. A) Boxplot and density plot showing the distribution of estimated values of epigenetic ages in Control and OAVS cohorts. Boxplots and density plots were created by using "Graphics" and "sm" packages, respectively. B) Boxplot of selected cellular components of OAVS patients and controls. For each panel, the thick horizontal line represents the median of the distribution while the box represents the interquartile range. Whiskers are set as the default option for the boxplot function and extend to the most extreme data point which is no more than 1.5 times the interquartile range from the box. Open circles represent single values exceeding 1.5 interquartile ranges.

Supplementary Figure S4

A)

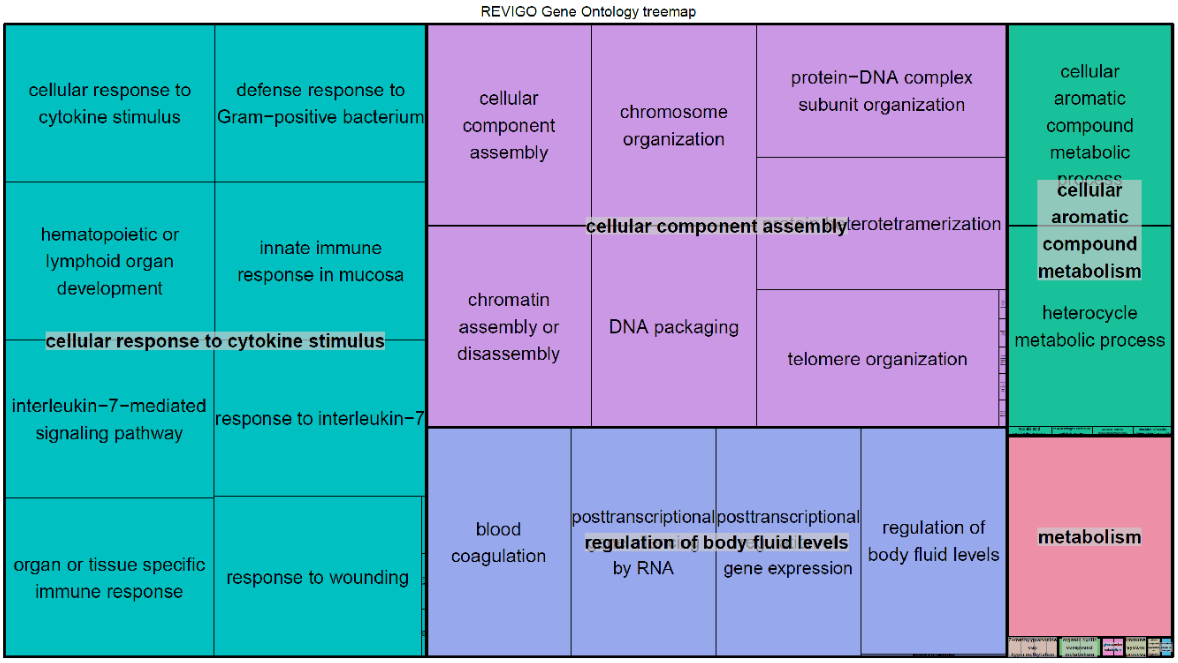

B)

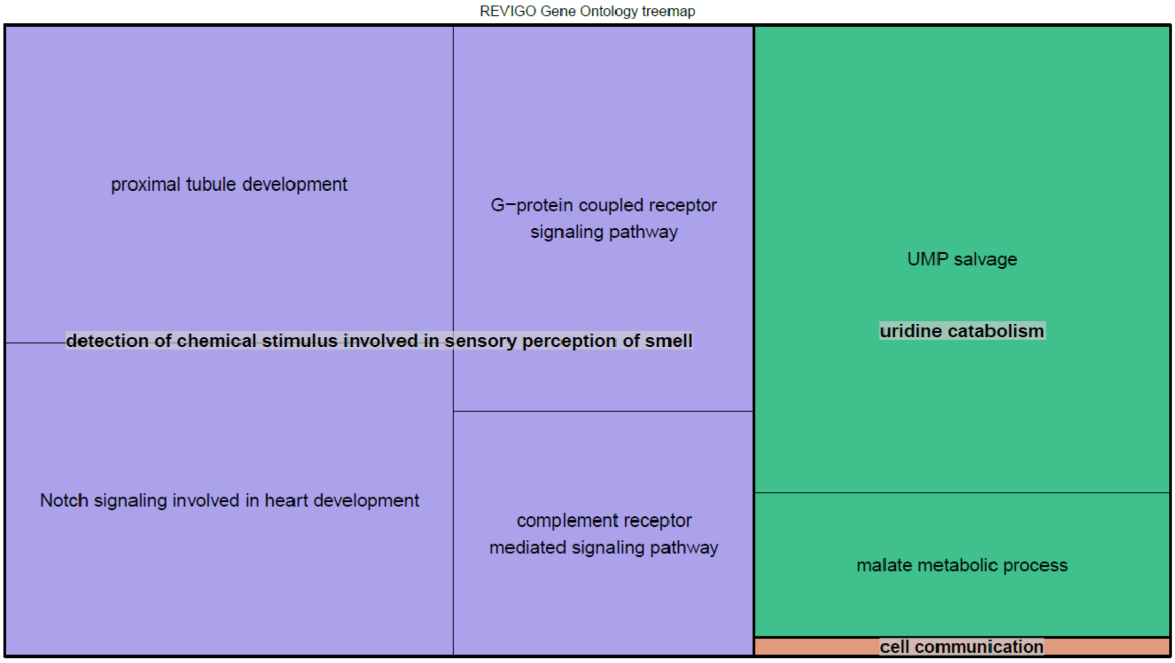

Figure S4. REVIGO treemaps of Gene Ontology (GO) biological processes enriched in hyper- (top panel A) and hypomethylated (bottom panel B) gene regions. For each panel, not all enriched terms are reported due to space constraints.

Supplementary Figure S5

.

Summary Network Barplot Details

Barplot

View source data (http://phenolyzer.wglab.org/done/38799/-tgXxHlvOQYJ7usU/index.htmlout.final_gene_list)

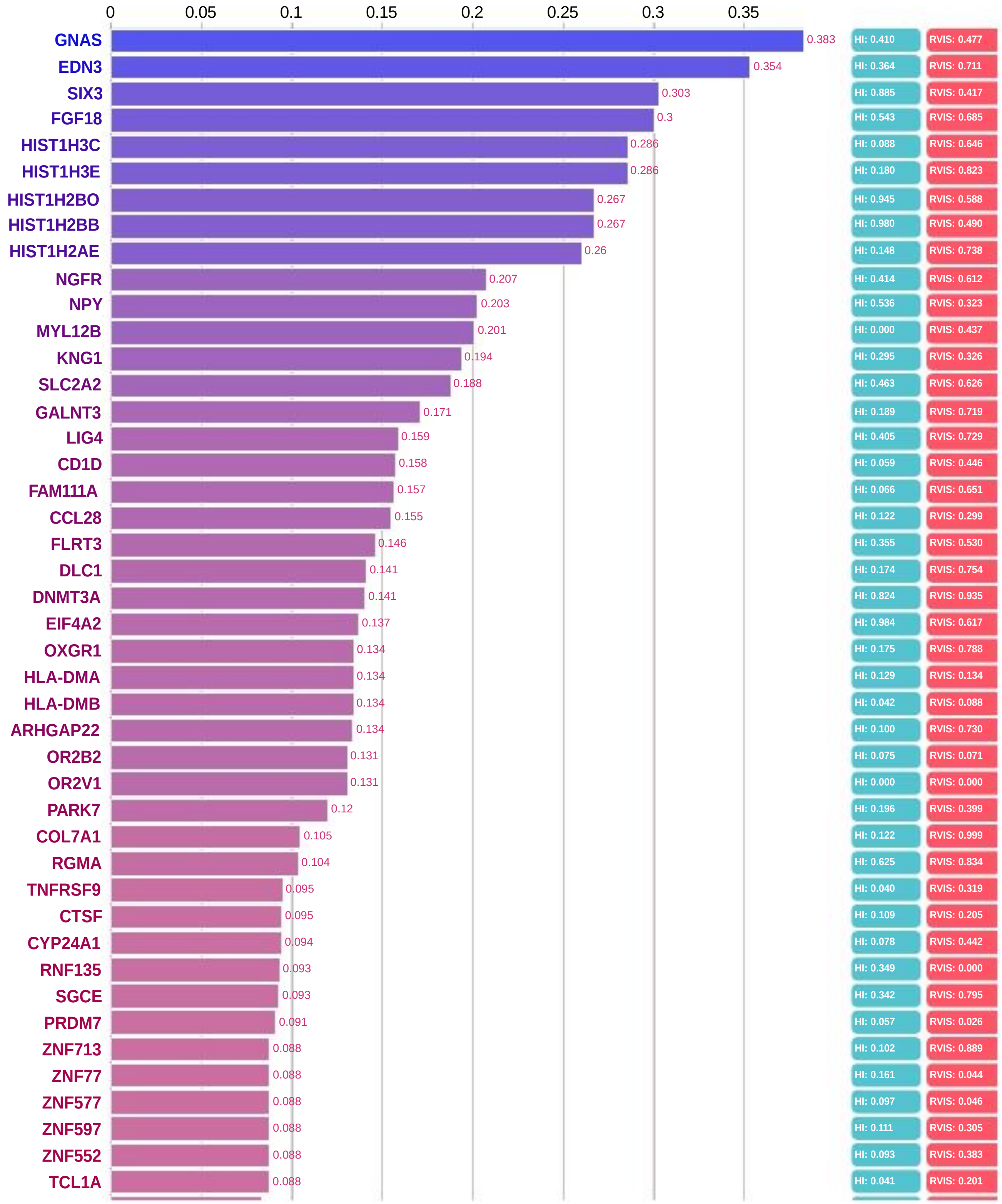

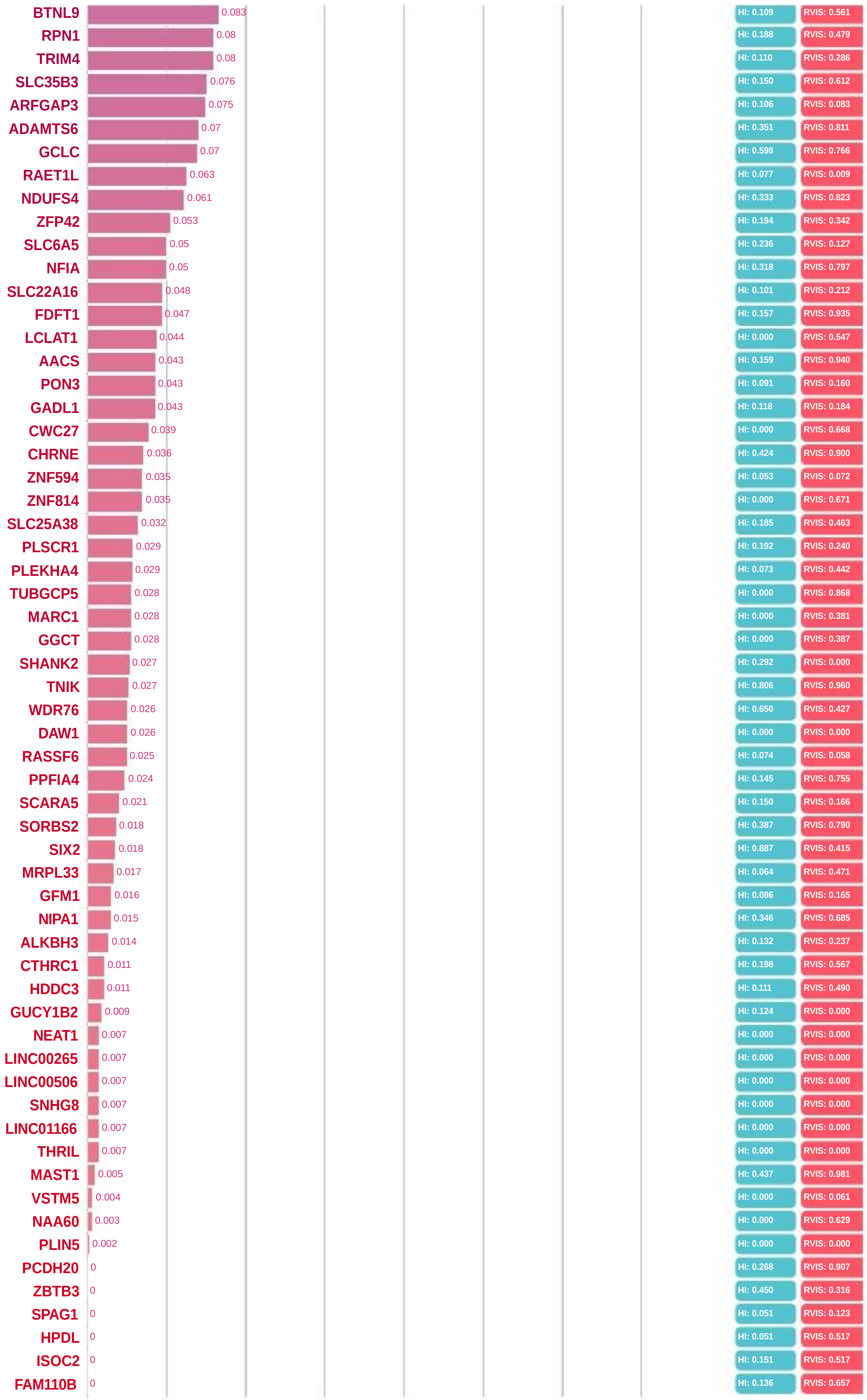
BTNL9 0.083 HI: 0.109 RVIS: 0.561
RPN1 0.08 HI: 0.188 RVIS: 0.479
TRIM4 0.08 HI: 0.110 RVIS: 0.286
SLC35B3 0.076 HI: 0.150 RVIS: 0.612
ARFGAP3 0.075 HI: 0.106 RVIS: 0.083
ADAMTS6 0.07 HI: 0.351 RVIS: 0.811
GCLC 0.07 HI: 0.598 RVIS: 0.766
RAET1L 0.063 HI: 0.077 RVIS: 0.009
NDUFS4 0.061 HI: 0.333 RVIS: 0.823
ZFP42 0.053 HI: 0.194 RVIS: 0.342
SLC6A5 0.05 HI: 0.236 RVIS: 0.127
NFIA 0.05 HI: 0.318 RVIS: 0.797
SLC22A16 0.048 HI: 0.101 RVIS: 0.212
FDFT1 0.047 HI: 0.157 RVIS: 0.935
LCLAT1 0.044 HI: 0.000 RVIS: 0.547
AACS 0.043 HI: 0.159 RVIS: 0.940
PON3 0.043 HI: 0.091 RVIS: 0.160
GADL1 0.043 HI: 0.118 RVIS: 0.184
CWC27 0.039 HI: 0.000 RVIS: 0.668
CHRNE 0.036 HI: 0.424 RVIS: 0.900
ZNF594 0.035 HI: 0.053 RVIS: 0.072
ZNF814 0.035 HI: 0.000 RVIS: 0.671
SLC25A38 0.032 HI: 0.185 RVIS: 0.463
PLSCR1 0.029 HI: 0.192 RVIS: 0.240
PLEKHA4 0.029 HI: 0.073 RVIS: 0.442
TUBGCP5 0.028 HI: 0.000 RVIS: 0.868
MARC1 0.028 HI: 0.000 RVIS: 0.381
GGCT 0.028 HI: 0.000 RVIS: 0.387
SHANK2 0.027 HI: 0.292 RVIS: 0.000
TNIK 0.027 HI: 0.806 RVIS: 0.960
WDR76 0.026 HI: 0.650 RVIS: 0.427
DAW1 0.026 HI: 0.000 RVIS: 0.000
RASSF6 0.025 HI: 0.074 RVIS: 0.058
PPFIA4 0.024 HI: 0.145 RVIS: 0.755
SCARA5 0.021 HI: 0.150 RVIS: 0.166
SORBS2 0.018 HI: 0.387 RVIS: 0.790
SIX2 0.018 HI: 0.887 RVIS: 0.415
MRPL33 0.017 HI: 0.064 RVIS: 0.471
GFM1 0.016 HI: 0.086 RVIS: 0.165
NIPA1 0.015 HI: 0.346 RVIS: 0.685
ALKBH3 0.014 HI: 0.132 RVIS: 0.237
CTHRC1 0.011 HI: 0.198 RVIS: 0.567
HDDC3 0.011 HI: 0.111 RVIS: 0.490
GUCY1B2 0.009 HI: 0.124 RVIS: 0.000
NEAT1 0.007 HI: 0.000 RVIS: 0.000
LINC00265 0.007 HI: 0.000 RVIS: 0.000
LINC00506 0.007 HI: 0.000 RVIS: 0.000
SNHG8 0.007 HI: 0.000 RVIS: 0.000
LINC01166 0.007 HI: 0.000 RVIS: 0.000
THRIL 0.007 HI: 0.000 RVIS: 0.000
MAST1 0.005 HI: 0.437 RVIS: 0.981
VSTM5 0.004 HI: 0.000 RVIS: 0.061
NAA60 0.003 HI: 0.000 RVIS: 0.629
PLIN5 0.002 HI: 0.000 RVIS: 0.000
PCDH20 0 HI: 0.268 RVIS: 0.907
ZBTB3 0 HI: 0.450 RVIS: 0.316
SPAG1 0 HI: 0.051 RVIS: 0.123
HPDL 0 HI: 0.051 RVIS: 0.517
ISOC2 0 HI: 0.151 RVIS: 0.517
FAM110B 0 HI: 0.136 RVIS: 0.657

Supplementary Figure S6

.

Summary Network Barplot Details

Barplot

View source data (http://phenolyzer.wglab.org/done/38800/pGNKnFwdAJc1l5ZT/index.htmlout.final_gene_list)

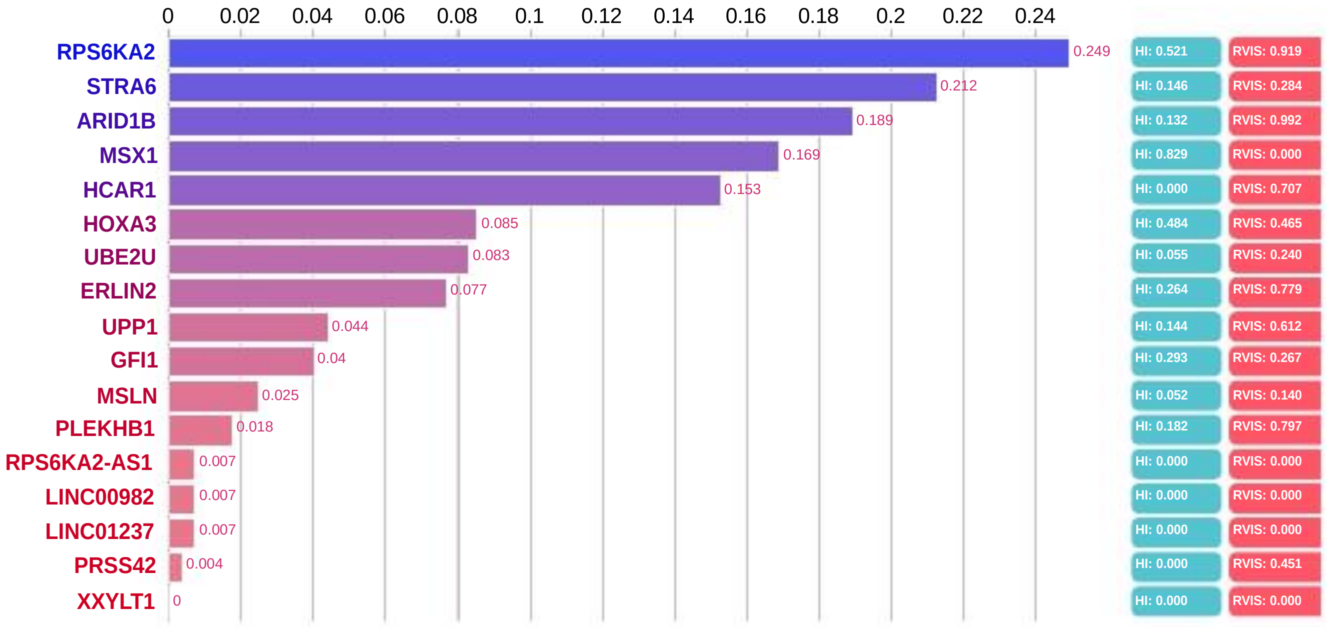

All Rights Reserved @Wang Genomics Lab 2010-2020
