# Supplementary material for: Genome-Wide DNA Methylation Analysis of a Cohort of 41 Patients Affected by Oculo-Auriculo-Vertebral Spectrum (OAVS)
Source: Int J Mol Sci. 2021 Jan 26;22(3):1190. doi: 10.3390/ijms22031190 (PMC7866060; doi:10.3390/ijms22031190)
Supplement: Supplementary file 1 [file ijms-22-01190-s001.zip › ijms-1072464 supplementary done/ijms-1072464_Suppl. Table S1.pdf]

Supplementary File 1: Table 1

Detailed clinical information of the 41 OAVS patients.

					Cranofacial						Ear						Ocular				Vertebral					Others					
OAVS Code	Sex	Age (years)	Familial OAVS	Prenatal factors	Hemifacial microsomia	Cleft lip/palate	Facial nerve weakness	Mandibular hypoplasia	Frontal bossing	Other craniofacial features	Preauricular skin tags/pits	Atresia of external auditory canal	Microtia/anotia	Hearing loss	Dysplastic ears	Asymmetric ears	Epibulbar dermoid	Microphthalmos	Coloboma upper eyelid	Other eye anomalies	Hemivertebrae	Fusion	Scoliosis	Kyphosis	Other vertebral anomalies	Cardiovascular	Brain	Developmental delay	Genitourinary malformation	Radial defects	Other organs/systems
**01**	M	na	-	ne	R<L	-	-	+	-	-	+	-	-	+	+	-	+	-	-	-	-	-	-	-	+	-	-	-	-	-	-
**02**	M	6	-	ne	L<R	-	-	+	-	-	+		-	+	+	-	-	-	-	-	-	+	-	-	-	-	-	-	-	-	-
**03**	M	na	+	ne	R<L	-	-	+	-	-	-	-	-	-	+	-	-	-	-	-	-	-	-	-	-	-	-	-	-	-	-
**04**	F	na	-	ne	L<R	-	-	+	-	+	+	-	+	-	+	-	-	-	-	-	-	-	-	-	-	-	-	+	-	-	+
**05**	F	5	-	-	R<L	-	-	+	-	-	+	-	-	+	+	-	-	+	-	-	-	-	-	-	+	+	-	-	-	-	-
**07**	F	42	-	-	R<L	-	-	-	-	-	+	-	-	-	+	+	-	+	-	+	-	-	-	-	-	ne	-	-	-	-	-
**08**	M	14	-	-	L<R	-	-	+	-	-	+	-	-	+	+	+	-	-	-	-	-	-	-	-	+	+	-	-	+	-	-
**09**	M	25	-	-	L<R	-	-	+	-	-	+	-	-	-	+	+	-	-	-	-	-	-	-	-	-	-	-	-	-	-	-
**10**	M	40	-	ne	R<L	-	-	+	-	+	+	-	-	-	-	+	+	-	-	-	-	-	-	-	-	-	-	-	-	-	-
**12**	F	17	-	-	L<R	-	-	+	-	-	+	-	-	-	+	-	+	-	-	-	-	-	-	-	-	-	-	-	-	-	-
**13**	F	3	+	-	L<R	-	-	+	-	-	+	-	-	+	+	-	-	-	-	+	-	-	-	-	-	+	ne	+	-	-	-
**14**	F	6	-	+	R<L	-	-	+	-	+	+	+	-	+	-	-	-	-	-	+	-	-	-	-	+	+	+	-	-	+	+
**15**	F	1	-	-	R<L	-	-	+	-	-	+	-	-	-	-	-	+	-	-	-	-	-	-	-	-	-	-	-	-	-	-
**16**	F	1	+	-	L<R	+	-	+	-	-	-	+	-	-	+	-	-	-	-	-	ne	ne	ne	ne	ne	+	-	-	-	-	-
**18**	M	7	-	-	L<R	-	-	+	-	-	+	-	-	-	+	-	+	-	-	-	-	-	-	-	-	-	-	-	-	-	-
**19**	M	8	-	ne	L<R	+	-	+	-	-	+	-	+	+	+	-	-	-	-	-	-	-	-	-	-	+	-	-	-	-	-
**20**	M	4	-	ne	R<L	+	-	+	-	-	+	+	+	+	+	-	+	-	-	-	+	-	-	-	-	+	+	+	-	-	-
**21**	M	10	-	ne	L<R	+	-	+	+	-	+	ne	-	ne	+	-	-	-	-	-	-	-	-	-	-	-	+	+	+	-	+
**22**	M	5	-	-	L<R	-	+	+	-	-	-	-	+	-	+	-	-	-	-	-	ne	ne	ne	ne	ne	+	+	+	-	-	-
**23**	F	8	-	-	L<R	-	-	+	-	-	+	-	-	-	-	-	+	-	-	-	+	-	+	-	+	-	-	-	+	+	-
**24**	M	30	-	-	L<R	-	-	+	-	-	+	+	-	+	+	-	-	+	-	-	-	-	-	-	-	-	+	-	+	-	-
**26**	F	1	-	ne	L<R	-	-	+	-	-	-	+	-	-	+	-	-	-	-	+	-	-	-	-	-	-	-	-	-	-	+

**27**	F	0.02	-	-	L<R	-	-	+	-	-	+	-	+	-	-	-	+	-	-	-	ne	ne	ne	ne	ne	+	-	-	-	-	-
**28**	M	24	-	-	L<R	-	-	+	-	-	+	-	-	-	+	-	+	+	+	-	ne	ne	ne	ne	ne	-	-	-	-	-	-
**29**	F	3	+	-	R<L	+	+	+	-	+	+	+	+	-	-	-	-	-	-	-	-	-	-	-	-	-	-	+	-	-	-
**30**	M	na	-	-	ne	-	-	+	-	-	+	-	-	-	-	-	-	-	-	-	-	-	-	-	+	+	-	-	-	-	-
**32**	F	6	-	-	L<R	-	-	+	-	-	+	+	-	+	+	-	-	-	-	-	-	-	-	-	-	+	-	-	-	-	+
**33**	M	7	+	+	R<L	-	-	+	-	-	-	+	-	+	+	-	+	-	-	-	+	+	+	-	+	+	-	-	+	+	-
**34**	M	20	-	-	R<L	-	-	-	-	-	-	-	+	+	+	-	+	+	+	+	-	-	-	-	-	-	-	-	-	-	-
**35**	M	4	-	-	R<L	-	-	+	-	-	+	-	-	+	+	-	-	-	-	-	-	-	-	-	-	+	-	-	-	-	-
**36**	F	1	-	ne	R<L	-	-	-	-	-	+	-	-	-	-	-	-	+	-	+	-	-	-	-	-	-	-	-	-	-	-
**37**	M	46	-	ne	L<R	-	-	-	-	-	+	-	+	+	+	-	-	+	-	-	-	-	-	-	-	-	-	-	-	-	-
**39**	M	6	-	ne	R<L	-	-	+	-	-	-	-	-	+	+	-	+	-	-	-	-	-	-	-	+	-	-	+	+	-	-
**40**	F	7	-	+	R<L	-	-	+	-	-	+	-	-	-	+	-	-	-	-	+	ne	ne	ne	ne	ne	-	-	-	-	-	-
**41**	F	14	-	-	R<L	-	-	+	-	-	+	-	-	+	+	-	-	+	-	+	-	-	-	-	+	+	+	+	-	-	+
**42**	F	0.58	-	-	R<L	+	-	+	-	-	+	-	-	-	-	-	+	+	-	+	-	-	-	-	-	-	+	ne	-	-	+
**43**	F	0.42	-	-	ne	-	-	+	-	-	+	-	-	-	-	-	-	-	+	-	-	-	-	-	-	-	-	-	-	-	-
**44**	M	9	-	+	L<R	-	-	+	-	-	+	+	+	+	+	+	-	-	-	-	-	-	-	-	-	-	-	-	-	-	-
**45**	F	1	-	ne	R<L	+	+	+	-	-	-	-	+	-	+	-	-	-	-	-	-	-	-	-	-	+	-	-	-	-	+
**46**	M	na	ne	ne	+	-	-	-	-	-	-	-	-	-	+	-	-	-	-	-	-	-	-	-	-	-	-	+	-	-	-
**47**	M	13	-	ne	R<L	+	-	+	-	-	-	-	-	+	+	-	-	-	-	-	+	-	-	-	+	-	-	-	+	-	-

Table legend: **na**=not available; **ne**=not evaluated; **L**=left; **R**=right
